# Long non-coding RNA linc00673 regulated non-small cell lung cancer proliferation, migration, invasion and epithelial mesenchymal transition by sponging miR-150-5p

**DOI:** 10.1186/s12943-017-0685-9

**Published:** 2017-07-11

**Authors:** Wei Lu, Honghe Zhang, Yuequn Niu, Yongfeng Wu, Wenjie Sun, Hongyi Li, Jianlu Kong, Kefeng Ding, Han-Ming Shen, Han Wu, Dajing Xia, Yihua Wu

**Affiliations:** 1 Department of Toxicology, Zhejiang University School of Public Health, 866 Yuhangtang Road, Hangzhou, People’s Republic of China; 2 Department of Surgical Oncology, Second Affiliated Hospital, Zhejiang University School of Medicine, Hangzhou, People’s Republic of China; 3 Department of Pathology, Zhejiang University School of Medicine, Hangzhou, People’s Republic of China; 4 Department of Physiology, Yong Loo Lin School of Medicine, National University of Singapore, Singapore, Singapore; 5 Department of Ophthalmology, Second Affiliated Hospital, Zhejiang University School of Medicine, Hangzhou, People’s Republic of China

**Keywords:** linc00673, miR-150-5p, Epithelial mesenchymal transition, Competing endogenous RNA, Non-small cell lung cancer

## Abstract

**Background:**

The function of a new long non-coding RNA linc00673 remains unclear. While identified as an oncogenic player in non-small cell lung cancer (NSCLC), linc00673 was found to be anti-oncogenic in pancreatic ductal adenocarcinoma (PDAC). However whether linc00673 regulated malignancy and epithelial mesenchymal transition (EMT) has not been characterized.

**Methods:**

Cell proliferation was assessed using CCK-8 and EdU assays, and cell migration and invasion were assessed using scratch assays and transwell invasion assays. Epithelial mesenchymal transition was examined using western blot, qRT-PCR and immunofluorescence staining. Interaction between miRNA and linc00673 was determined using luciferase reporter assays. In vivo experiments were performed to assess tumor formation. In addition, the expression data of NSCLC specimens of TCGA and patient survival data were utilized to explore the prognostic significance of linc00673.

**Results:**

In the present study, we found high linc00673 expression was associated with poor prognosis of NSCLC patients. In vitro experiments showed linc00673 knockdown reversed TGF-β induced EMT, and miR-150-5p was predicted to target linc00673 through bioinformatics tools. Overexpression of miR-150-5p suppressed lin00673’s expression while inhibition of miR-150-5p led to significant upregulation of lin00673, suggesting that linc00673 could be negatively regulated by miR-150-5p, which was further confirmed by the inverse correlation between linc00673 and miR-150-5p in NSCLC patients’ specimen. Furthermore, we proved that miR-150-5p could directly target linc00673 through luciferase assay, so linc00673 could sponge miR-150-5p and modulate the expression of a key EMT regulator ZEB1 indirectly. In addition, miR-150-5p inhibition abrogated linc00673 silence mediated proliferation, migration, invasion and EMT suppressing effect. Moreover, the inhibition of linc00673 significantly attenuated the tumorigenesis ability of A549 cells in vivo.

**Conclusions:**

We validated linc00673 as a novel oncogenic lncRNA and demonstrated the molecular mechanism by which it promotes NSCLC, which will advance our understanding of its clinical significance.

**Electronic supplementary material:**

The online version of this article (doi:10.1186/s12943-017-0685-9) contains supplementary material, which is available to authorized users.

## Background

The ENCODE program has elucidated that about 90% of human genome DNA sequence is actively transcribed, however only 2% of those transcripts encode proteins, while vast remaining transcripts are termed as non-coding RNAs (ncRNAs) [1–3]. MicroRNAs (miRNAs) and long non-coding RNAs (lncRNAs) constitute the majorities of ncRNAs. MiRNAs are evolutionarily conserved single-stranded RNAs containing about 21–24 nucleotides. MiRNAs are involved in numerous biological processes and play a critical role in mRNA post-transcriptional regulation by targeting 3′ untranslated regions (UTRs) of mRNAs with their seed sequences (2–7 nucleotides in the 5′ end), resulting in mRNA degradation or translation inhibition [4, 5]. lncRNAs are another type of regulatory ncRNAs, the length of which usually exceed 200 nt without protein-coding capacity [6]. Despite that lncRNAs were once considered as transcriptional noise, they have now been demonstrated to participate in a wide variety of epigenetic regulatory processes, such as histone modification, chromatin remodeling, transcriptional regulation and RNA alternative splicing [7, 8].

Lung cancer is one of the major causes of cancer-related death worldwide, which is classified into small cell lung cancer (SCLC) and non-small cell lung cancer (NSCLC) [9]. Despite the fact that great progress has been made in lung cancer diagnosis and treatment, 57% of lung cancers are diagnosed at a distant stage due to its typically asymptomatic early stage, and the 5-year survival rate for NSCLC still remain at less than 20% [9, 10]. Metastasis is the primary characteristic of cancer and is the leading cause of death for about 90% of cancer patients. Complicated signaling pathways were involved in tumor metastasis. Among them epithelial mesenchymal transition (EMT) has become the classical tumor metastasis theory in recent years [11]. During the EMT process, epithelial cells lose adhesion and apical-basal polarity, change cytoskeleton composition and acquire phenotypes of mesenchymal cells or fibroblasts such as increased migration ability and invasiveness, and then invade adjacent tissues and metastasize to distant organs [12–14]. TGF-β and TNF-α are crucial cytokines during the EMT process. Canonical TGF-β signaling pathway could activate EMT-associated transcriptional factors such as Snail, Twist and ZEB1 via Smad signaling [14, 15]; TNF-α could enhance mesenchymal phenotype via activating NF-κB/Snail signaling pathway [16, 17]. Currently, numerous lncRNAs such as lncRNA-ATB and MALAT1 have been reported to be involved in the EMT process in liver cancer and bladder cancer, respectively [18, 19]. Another lncRNA ZEB1-AS1 has been shown to associate with EMT process and poor prognosis in hepatocellular carcinoma [20]. Upregulation of lncRNA HOTTIP has been proved to induce EMT and promote metastasis of esophageal squamous cell carcinoma [21]. MEG3 lncRNA has been found to play a critical role in the TGF-β induced EMT in lung cancer [22]. But up to now, the molecular mechanisms of lncRNAs mediated EMT remains largely unknown.

Linc00673 is a new lncRNA identified to be oncogenic in NSCLC but anti-oncogenic in PDAC, which seemed to be controversial [23, 24]. Furthermore, both of the two studies focused on the cancer cell’s proliferation regulated by linc00673, but the regulatory mechanisms of EMT was still unknown. Recently, the “competing endogenous RNA (ceRNA) hypothesis” has been raised [25], which has been already validated by a plenty of studies in many different biological events, such as muscle differentiation, tumor suppression and drug resistance [26–28]. With the rapid identifications of lncRNAs, many of them have been shown to act as ceRNAs through sequestrating target miRNAs and regulating miRNA target genes indirectly. Some studies have reported various lncRNAs acting as ceRNAs to regulate the EMT process, such as lncRNA HULC in hepatocellular carcinoma and lncRNA H19 in colorectal cancer [29, 30]. A plenty of miRNAs were reported to either suppress or promote the EMT process by targeting key EMT-regulators such as ZEB1, Snail, Twist and E-cadherin [31]. Thus the investigation on whether linc00673 acted as an EMT-associated ceRNA seems to be promising.

In the present study, we confirmed the oncogenic role of linc00673 which may serve as an effective prognostic marker for NSCLC patients. We observed that linc00673 knockdown reversed TGF-β induced epithelial mesenchymal transition through in vitro and in vivo experiments. We demonstrated that linc00673 modulated cell proliferation, migration, invasion and EMT by sponging miR-150-5p and regulating ZEB1 expression indirectly. Besides, miR-150-5p inhibition abrogated linc00673 silence mediated proliferation, migration, invasion and EMT suppressing effect.

## Methods

### Chemicals and cell lines

Recombinant human TGF-β and TNF-α were purchased from PeproTech and stored as 10 ng/μL stocking at −20 °C. TGF-β receptor antagonist SB-431542 was purchased from Cayman Chemical and store as 5 mM stocking at −20 °C.

A human bronchiolar epithelial cell line (BEAS-2B), six human non-small-cell lung cancer cell lines (A549, H1975, H596, H520, H1650, H1703) and HEK-293 T cell line were obtained from Chinese Academy of Sciences. HEK-293 T was cultured in DMEM medium (Gibco) and other cell lines were cultured in RPMI 1640 medium (Gibco) supplemented with 10% fetal bovine serum at 37 °C in a humidified atmosphere containing 5% CO_2_. Cells in the logarithmic growth phase were used in the subsequent experiments.

### siRNA, miRNA and plasmid DNA transfection

Negative control siRNA, siRNAs targeting linc00673, miRNA mimics and inhibitors were designed and synthesized by Genepharma. The siRNA sequences were listed in Additional file 1: Table S1. The over-expression vector of linc00673 was constructed in pcDNA3.1 (+) by Genscript. The pmirGLO Dual-Luciferase miRNA Target Expression Vector was purchased from Promega. Transfection of siRNA, miRNA or plasmid DNA was performed using PowerFect in vitro siRNA transfection reagent (SignaGen) according to the manufacturer’s recommendations.

### Western blot

The cells were collected and lysed with cell lysis buffer (Beyotime). Samples of the lysates were separated on 6%–15% SDS-PAGE gels and transferred to nitrocellulose filter membranes. The membranes were incubated with primary antibodies at 4 °C overnight, including Vimentin (1:1000, Cell signaling Technology, CST), E-cadherin (1:500, Santa Cruz), N-cadherin (1:500, CST), ZEB1 (1:500, CST), Snail (1:500, CST), PARP (1:1000, CST), pro-caspase 3 (1:1000, CST), GAPDH (1:2000, CST), followed by incubation with an HRP-conjugated anti-mouse or anti-rabbit secondary antibody separately. Finally, the bands were detected by ChemiScope 3300 Mini (Clinx) using the ECL substrate (Cyanagen).

### RNA extraction and quantitative real-time PCR

Total RNA was extracted from cells using Trizol reagent (Sangon Biotech) according to the manufacturer’s instructions. For mRNAs and lncRNA quantification, RNA was reverse transcribed to cDNA using PrimeScript™ RT reagent Kit with gDNA Eraser (Takara). Quantitative real-time PCR was performed using cDNA primers specific for mRNA or lncRNA. The gene GAPDH was used as an internal control. For miRNA quantification, reverse transcription was performed using Mir-X™ miRNA First Strand Synthesis Kit (Takara). MiRNA specific 5′ primers and mRQ 3′ primer was used during quantitative real-time PCR. The gene U6 was used as an internal control. Primer sequences were provided in Additional file 2: Table S2. All the real-time PCR reactions were performed using Takara’s SYBR Premix Ex Taq™ II (Tli RNaseH Plus) in Applied Biosystems 7500 Fast Real-Time PCR System (Applied Biosystems). The 2^-△△Ct^ method was used for quantification and fold change for target genes was normalized by internal control.

### EdU and CCK-8 proliferation assay

The EdU (5-ethynyl-2′-deoxyuridine) proliferation assay was performed using Cell-Light EdU Apollo 567 In Vitro Imaging Kit (Ribobio) following the manufracture’s recommendation. In brief, cells were exposed to the indicated treatments accordingly, and then approximately 5*10^3^ cells/wells were seeded into 96-well plates. 24 h after seeded, 100 μl medium containing 50 μM EdU was added into each well and cells were incubated for 2 h at 37 °C, fixed with 4% paraformaldehyde, then stained with Hoechst 33,342 and Apollp reaction cocktail. Images were captured using a fluorescence microscopy (Nikon) and merged using Adobe Photoshop 6.0 software. Afterwards EdU-positive cells and total cells were counted within each field.

The cell proliferation was also assessed using the CCK-8 assay (Boster) according to the manufacturer’s protocol. To be brief, approximately 2*10^3^ cells were plated into 96-well plates. After cells adhered, 10 μl CCK-8 solution was added to each well and incubated at 37 °C for 1 h. The cell proliferation curves were plotted by measuring 450 nm absorbance at each indicated time point. Experiments were performed in triplicate.

### Colony forming assay

Cells were exposed to the indicated treatments accordingly and were seeded into 6 cm dishes (10^3^ cells/wells). After cultured for 14 days, colonies were fixed by 4% paraformaldehyde and stained by incubation with 0.4% crystal violet solution and the images were captured by a camera. Experiments were performed in triplicate.

### Flow cytometric analysis

Apoptosis assay was performed using FITC Annexin V Apoptosis Detection Kit I (BD Biosciences). Briefly, cells were harvested using trypsin, washed twice with ice-cold phosphate-buffered saline (PBS) and resuspended in 1*Binding Buffer at a concentration of 1*10^6^ cells/ml. 100 μl solution was transferred into a 5 mL culture tube then 5 μl PI and 5 μl FITC Annexin V were added. After incubated for 15 min at 25 °C in the dark, 400 μl 1*Binding Buffer was added to each tube and stained cells were analyzed by FACSCalibur Flow Cytometer (BD Biosciences).

### Immunofluorescence staining

Cells were seeded into 6-well plates and grown on sterilized coverslips. After exposed to the indicated treatments respectively, cells were fixed by 4% paraformaldehyde and permeabilized with 0.5% Triton X-100 for 10 min at room temperature. Next cells were blocked with 5% BSA (Amersco) in PBST for 1 h and incubated with primary antibodies against Vimentin (1:100, CST) at 4 °C overnight, then incubated with fluorochrome-labeled anti-rabbit secondary antibody (MultiSciences) for 1 h at room temperature. Subsequently, the coverslips were stained with DAPI (1:5000, Beyotime) and imaged using a fluorescence microscopy (Nikon). Images were merged using Adobe Photoshop 6.0 software.

### Transwell invasion assay and wound scratch assay

Transwell invasion assay was performed using 8.0 μm Transwell Permeable Supports (Corning). Cells were harvested 24 h after transfection and 5*10^4^ cells suspended in 100 μl serum-free medium were seeded into the upper chamber pre-coated with Matrigel Matrix (BD Biosciences), and 600 μl medium containing 10% FBS was added to the lower chamber. After incubation for 24 h, cells that did not invaded through the membrane were mechanically removed with a cotton swab. Next, 4% paraformaldehyde was used to fix the cells on the bottom surface of the membrane for 10 min, and then cells were stained with a 0.4% crystal violet solution. The invading cells were imaged using a digital microscopy (Nikon).

For wound scratch assay, cells were seeded at 3*10^5^cells/well in 6-well plates and exposed to treatments accordingly. After cells reached 100% confluence, a sterilized 200 μl pipette tip was used to make a straight scratch in the wells. Images were captured by a digital microscopy at each indicated time.

### Luciferase reporter assay

Cells (A549 and 293 T) were seeded at 5 × 10^4^ cells/well in 24-well plates and allowed to settle overnight. In the next day, cells were co-transfected with pmirGLO-linc00673-WT, pmirGLO-linc00673-MUT, pmirGLO-ZEB1–3’UTR-WT or pmirGLO-ZEB1–3’UTR-MUT reporter plasmids and mimics NC, miR-150-5p mimics accordingly. 24 h post transfection, cells were lysed using passive lysis buffer (promega) and the luciferase activity was measured by GloMax 20/20 Luminometer (Promega) using the Dual-Luciferase Reporter Assay System (Promega) and normalized to renilla luciferase activity respectively. Experiments were performed in triplicate.

### In vivo experiments

A549 cells stably transfected with linc00673-shRNA or negative control vector were constructed in our laboratory. All the transfected A549 cells were then labeled with pHIV-Luciferase. Next 8 NOD/SCID mice were divided into two groups and were injected with 1 × 10^6^/0.1 ml linc00673-shRNA or negative control shRNA (sh-nc) transfected A549 cells by tail vein injection, respectively. Bioluminescent flux (photons/s/sr/cm^2^) was determined every 2 weeks to assess tumor foci in lungs using IVIS spectrum imaging system (Caliper, Newton, USA) and Living Image software (Caliper, Newton, USA). The mice were sacrificed 35 days after injection.

### Computational analyses and bioinformatics

The linc00673 and miRNA expression data of NSCLC specimens of TCGA was extracted from exon expression dataset download from UCSC Cancer Browser (https://genome-cancer.ucsc.edu/, 2016/08/21), which was a suite of web-based tools to visualize, integrate and analyze cancer genomics and its associated clinical data. The quantification of linc00673’s expression was done by averaging the expression of its exons. Potential target miRNAs of linc00673 were predicted by the computer algorithm miRanda (http://www.microrna.org/microrna/home.do, 2016/08/21). The mature miRNA sequences used by miRanda was download from miRBase (http://www.mirbase.org/, 2016/08/21).

### Statistical analyses

Statistical analyses were performed using Graphad Prism 6 software or SPSS 19.0 software. Data of experiments are expressed as mean ± standard deviation (SD) of at least three independent experiments. Differences between two groups were assessed using Student’s *t*-test. We adopted one-way analysis of variance for multiple comparisons. The miRNAs with miRanda max scores were adopted for further prediction by spearman correlation and cox regression using TCGA dataset. The miRNAs which had significant negative correlation with linc00673 and were related to negative prognosis were considered potential linc00673 targeted miRNA candidates for further verification. Overall survival (OS) curves were constructed using the Kaplan–Meier method and the log-rank test was performed. A *p*-value < 0.05 was considered statistically significant unless additionally specified.

## Results

### Linc00673 is associated with poor survival in NSCLC patients

**Fig. 1:**
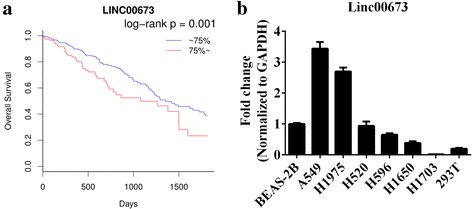
Linc00673 is associated with poor survival in NSCLC patients. **a**. Association of linc00673 expression with overall survival of NSCLC patients, red: expression above upper quartile, blue: expression below upper quartile. **b**. Linc00673 expression in 6 NSCLC cell lines and 1 human bronchial epithelial cell line as determined by qRT-PCR

Analysis of TCGA data demonstrated that higher linc00673 RNA level in NSCLC patients was correlated with worse overall survival outcome (Fig. 1a and Additional file 3: Figure S1), which implied that linc00673 served as a potential oncogene and had powerful prognostic value for NSCLC patients. We then detected linc00673 expression level in 6 NSCLC cell lines and 1 human bronchial epithelial cell line by performing qRT-PCR analysis, and we found 2 lung adenocarcinoma cell lines A549 and H1975 expressing high level linc00673 and 1 lung squamous carcinoma cell line H1703 expressing low level linc00673 (Fig. 1b).

### Effects of linc00673 on lung cancer cell apoptosis, viability, migration and invasion

**Fig. 2:**
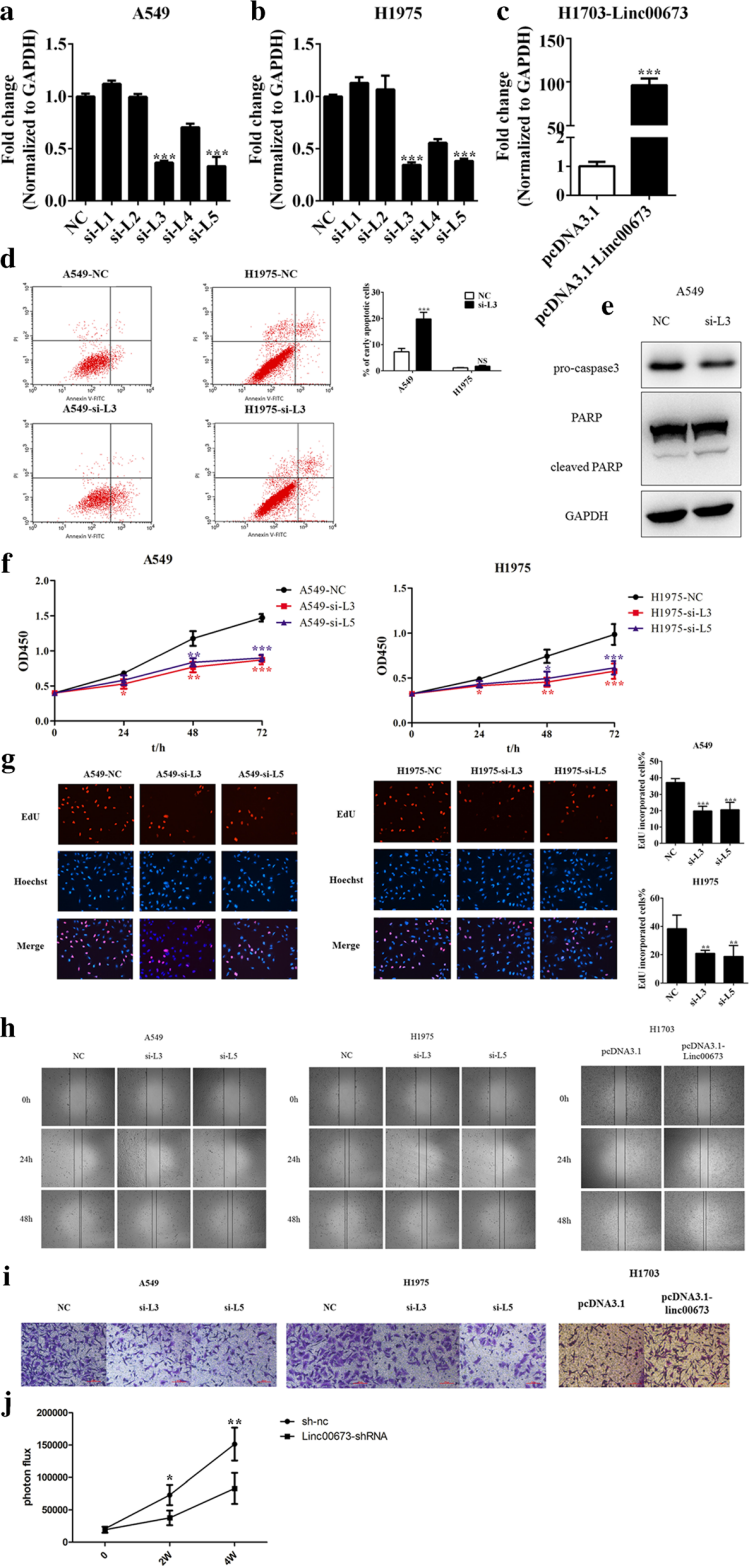
Effects of linc00673 on lung cancer cell apoptosis, viability, migration and invasion. **a**. Validation of siRNA knockdown efficiency in A549 cells as determined by qRT-PCR. **b**. Validation of siRNA knockdown efficiency in H1975 cells as determined by qRT-PCR. **c**. Validation of pcDNA3.1-linc00673 over-expression vector in H1703 cells as determined by qRT-PCR. **d**. Flow cytometric analysis of apoptosis in si-NC or si-L3 transfected A549 and H1975 cells. **e**. Expression of PARP and pro-Caspase 3 in si-NC or si-L3 transfected A549 cells as determined by western blot. **f**. CCK-8 proliferation assay in si-NC or si-L3/si-L5 transfected A549 and H1975 cells. **g**. EdU proliferation assay in si-NC or si-L3/si-L5 transfected A549 and H1975 cells. **h**. Wound scratch assay in si-NC or si-L3/si-L5 transfected A549 and H1975 cells and in pcDNA3.1-linc00673 transfected H1703 cells. **i**. Transwell invasion assay in si-NC or si-L3/si-L5 transfected A549 and H1975 cells and in pcDNA3.1-linc00673 transfected H1703 cells. **j**. Tumor growth in NOD/SCID mice with tail vein injection of linc00673-shRNA or sh-nc transfected A549 cells. Error bars indicate the mean ± SD. *p < 0.05, **p < 0.01, ***p < 0.005

To further investigate the role of linc00673 in the tumorigenesis of NSCLC, we used 2 linc00673 high expression cell lines A549, H1975 and one linc00673 low expression cell line H1703 in the following experiments. We transfected siRNA specific for linc00673 in A549 and H1975 cells and detected linc00673 expression level 48 h post transfection. As shown in Fig. 2a and b, the knockdown efficiency was best using si-L3 and si-L5. In order to reduce possibility of observation of off-target effects, we used these two distinct siRNAs to conduct following experiments. Meanwhile, we constructed linc00673 over-expression vector using pcDNA3.1, and linc00673 expression level was significantly elevated after transfection of pcDNA3.1-linc00673 in H1703 cells (Fig. 2c).

A multitude of lncRNAs participate in a broad range of biological processes. For instance, MALAT1 could modulate cell proliferation, invasion and apoptosis in various types of cancers [32–34]. Therefore we performed the following assays to explore the biological function of linc00673 in NSCLC. By performing apoptosis assay, we observed that knockdown linc00673 increased apoptosis slightly in A549 cells (Fig. 2d). Consistently, cleaved PARP increased while pro-Caspase 3 decreased after linc00673 knockdown in A549 cells (Fig. 2e). However, linc00673 knockdown did not increase apoptosis in H1975 cells (Fig. 2d and Additional file 4: Figure S2A), which was consistent with previous study [24]. CCK-8 proliferation assays were performed to detect cell viability, and we observed that the cell viability reduced significantly in si-L3 and si-L5 transfected cells compared to si-NC transfected cells (Fig. 2f and Additional file 4: Figure S2B). Similar growth inhibiting effect was also validated by EdU proliferation assays, where linc00673 knockdown reduced EdU incorporated cell proportion (Fig. 2g). Beyond that, linc00673 knockdown suppressed colony forming ability of A549 and H1975, as shown in Additional file 4: Figure S2C.

Tumor metastasis and invasion are the most life threatening aspects of lung cancer. We then examined whether linc00673 affected NSCLC cell migration and invasion abilities. By performing wound scratch assay in A549 and H1975, we found wound closure area was larger after linc00673 knockdown (Fig. 2h). Transwell invasion assay also showed decreased cell invasion ability after linc00673 siRNA transfection (Fig. 2i). Conversely, over-expression of linc00673 promoted cell migration and invasion in H1703 cells (Fig. 2h and i). We further validated our results in vivo. A549 cells transfected with linc00673-shRNA or sh-nc (negative control shRNA) were injected to NOD/SCID mice via tail veins. The tumor formation were significantly inhibited after linc00673 knockdown (*P* = 0.011 at week 2 and *P* = 0.008 at week 4; Fig. 2j and Additional file 5: Figure S3). The above results suggested linc00673 participate in a series of biological processes.

### Linc00673 was required for epithelial mesenchymal transition

**Fig. 3:**
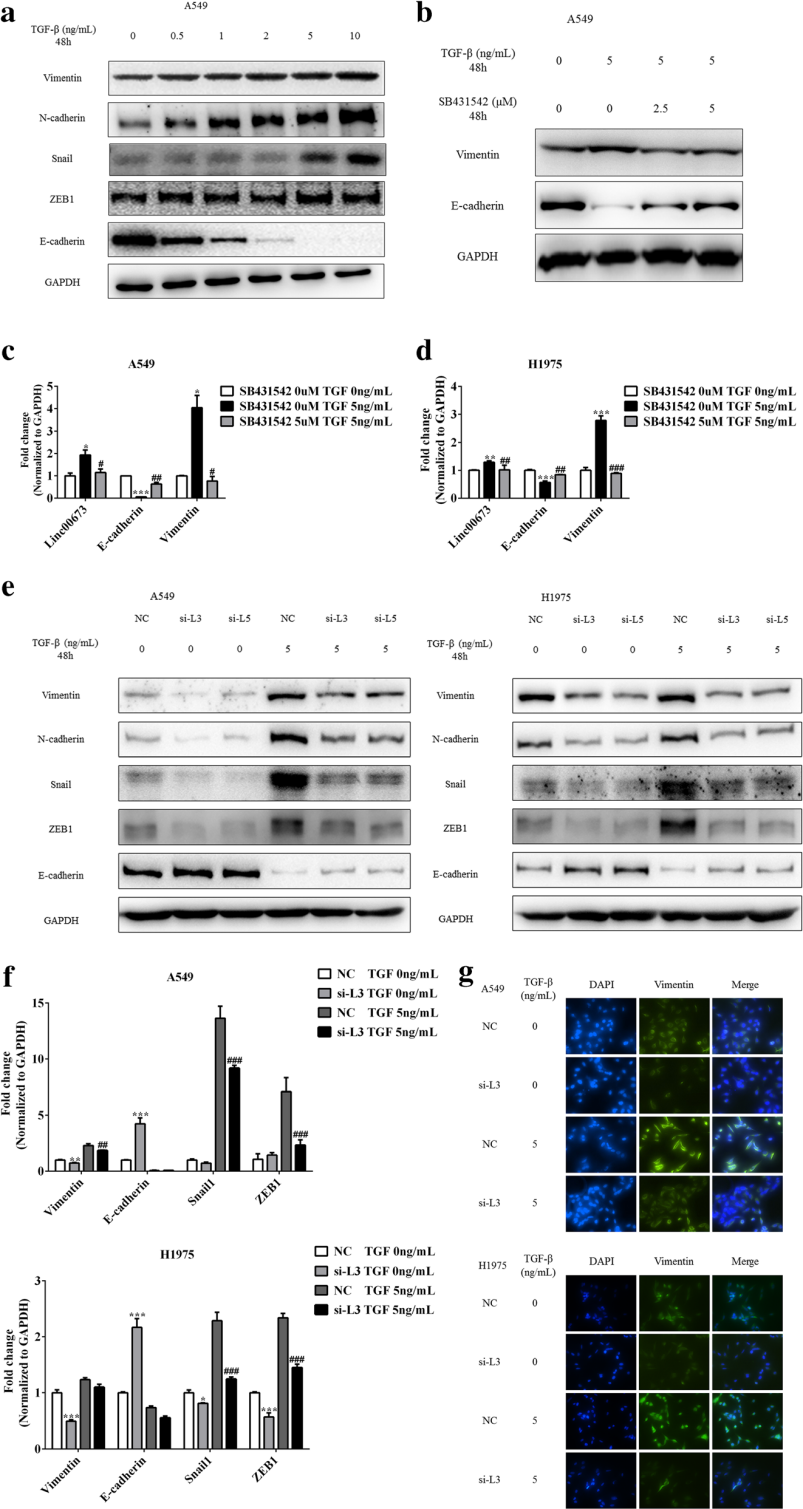
Linc00673 was required for epithelial mesenchymal transition. **a**. Expression of Vimentin, N-cadherin, Snail, ZEB1 and E-cadherin in TGF-β treated A549 cells as determined by western blot. **b**. Expression of Vimentin and E-cadherin in TGF-β receptor antagonist SB431542 and TGF-β treated A549 cells as determined by western blot. **c**. Expression of linc00673 in TGF-β and SB431542 treated A549 cells as determined by qRT-PCR. **d**. Expression of linc00673 in TGF-β and SB431542 treated H1975 cells as determined by qRT-PCR. **e**. Expression of EMT markers in si-NC or si-L3/si-L5 transfected followed by TGF-β treated A549 and H1975 cells as determined by western blot. **f**. mRNA level of EMT markers in si-NC or si-L3 transfected followed by TGF-β treated A549 and H1975 cells as determined by qRT-PCR. **g**. Immunofluorescence staining of Vimentin expression in si-NC or si-L3 transfected followed by TGF-β treated A549 and H1975 cells. Error bars indicate the mean ± SD. *p < 0.05, **p < 0.01, ***p < 0.005, #*p* < 0.05, ##*p* < 0.01, ###*p* < 0.005

Since linc00673 was positively associated with mesenchymal markers and negatively associated with epithelial markers in NSCLC tissues, we then investigated the role of linc00673 in the EMT process. TGF-β signaling was considered to play a pivotal role in regulating EMT and the TGF-β induced EMT model has been adopted in various cancer types [15, 18, 19], thus we used TGF-β to induce EMT in NSCLC cells. We observed that 5 ng/mL TGF-β was sufficient to induce EMT (Fig. 3a and Additional file 6: Figure S4A), while 5 uM TGF-β receptor antagonist SB431542 could block the EMT phenotypes in A549 and H1975 cells (Fig. 3b and Additional file 6: Figure S4B). In A549 cells, linc00673 expression was significantly elevated under the stimuli of TGF-β, which attenuated by SB431542 (Fig. 3c). However, in H1975 cells linc00673 increased slightly under TGF-β stimuli (Fig. 3d). These results suggested that linc00673 may participate in TGF-β induced EMT process in NSCLC.

Then we transfected siRNA specific for linc00673 followed by TGF-β stimulation. Knockdown of linc00673 changed TGF-β induced mesenchymal and spindle-like morphology in A549 and H1975 cells (Additional file 6: Figure S4C). Moreover, we observed that epithelial marker E-cadherin increased which was accompanied with the decrease of mesenchymal markers Vimentin, N-cadherin, Snail1 and ZEB1 following linc00673 depletion in A549 and H1975 cells (Fig. 3e and f). Consistent with this result, overexpression of linc00673in H1703 cells promoted EMT (Additional file 6: Figure S4D).

We also induced EMT using TNF-α and linc00673 knockdown reversed TNF-α induced EMT (Additional file 6: Figures. S4E and S4F), while linc00673 expression did not alter under TNF-α stimuli in A549 cells (Additional file 6: Figure S4G). Immunofluorescence staining revealed that linc00673 knockdown changed Vimentin distribution in the absence or presence of TGF-β in A549 and H1975 cells (Fig. 3g), while over-expression of linc00673 in H1703 cells showed opposite effect (Additional file 6: Figure S4H).

### Reciprocal correlation between linc00673 and miR-150-5p

**Fig. 4:**
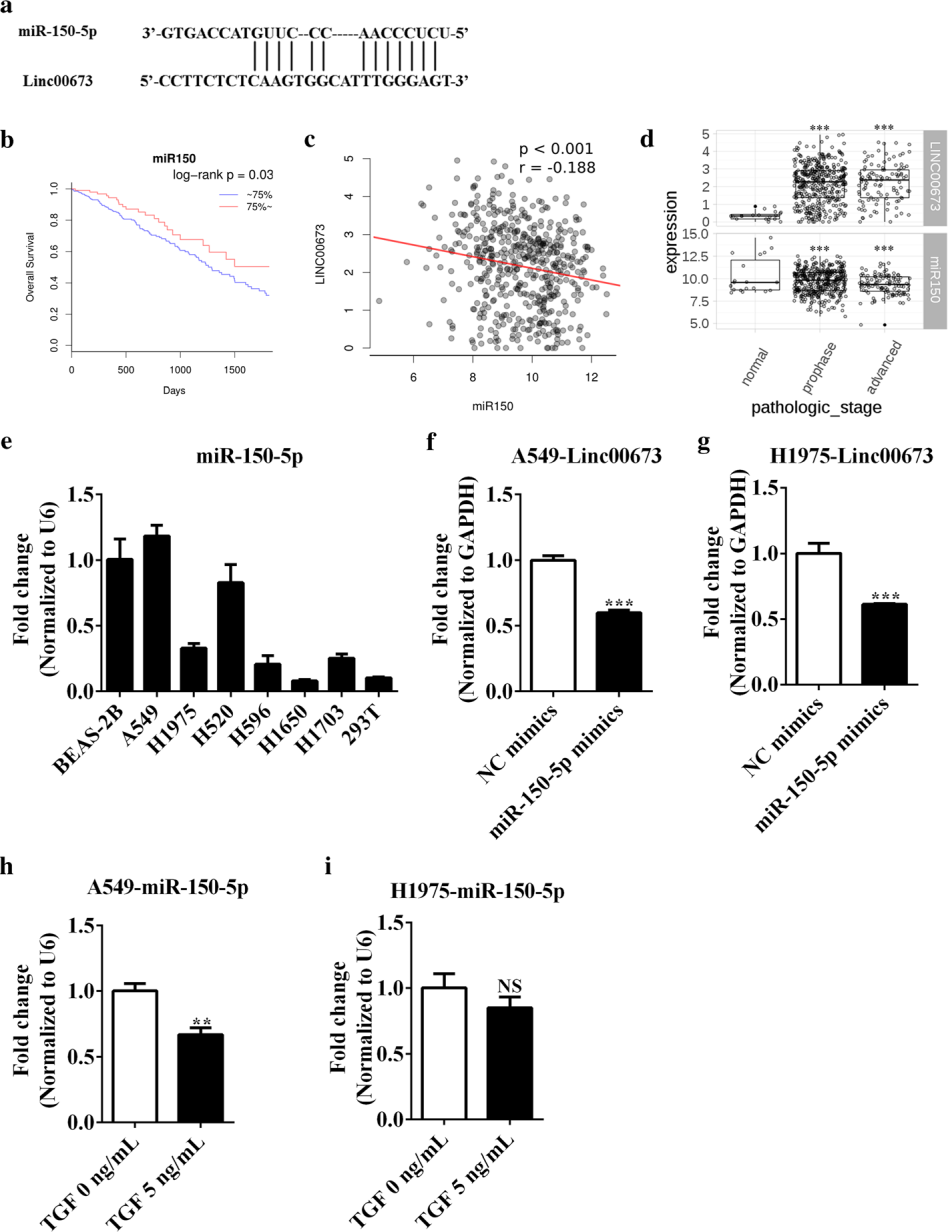
Reciprocal correlation between linc00673 and miR-150-5p. **a**. The predicted binding sites of miR-150-5p to the linc00673 sequence. **b**. Association of miR-150-5p expression with overall survival of NSCLC patients, red: expression above upper quartile, blue: expression below upper quartile. **c**. Negative correlation between linc00673 and miR-150-5p expression in NSCLC patients. **d**. Association of linc00673 and miR-150-5p expression with pathologic stage in NSCLC patients. “Prophase” stands for stage I/II and “advanced” stands for stage III/IV. **e**. MiR-150-5p expression in 6 NSCLC cell lines and 1 human bronchial epithelial cell line as determined by qRT-PCR. **f**. Expression of linc00673 in miR-150-5p mimics transfected A549 cells as determined by qRT-PCR. **g**. Expression of linc00673 in miR-150-5p mimics transfected H1975 cells as determined by qRT-PCR. **h**. Expression of miR-150-5p in TGF-β treated A549 cells as determined by qRT-PCR. **i**. Expression of miR-150-5p in TGF-β treated H1975 cells as determined by qRT-PCR. Error bars indicate the mean ± SD. *p < 0.05, **p < 0.01, ***p < 0.005

Using the human miRNA targets prediction tool miRanda combined with the data of TCGA, we predicted linc00673 could be targeted by several miRNAs (miR-29c-3p, miR-30c-2-3p and miR-150-5p). Among them, miR-150-5p got the highest score and has been proved to be involved in regulating the EMT process by regulating ZEB1 [35–37]. The predicted binding sites of miR-150-5p to the linc00673 sequence were illustrated in Fig. 4a. There was a significantly inverse correlation between linc00673 level and miR-150-5p level in NSCLC tissues, and higher miR-150-5p level predicted better overall survival outcome accordingly (Fig. 4b, c and Additional file 7: Figure S5). In addition, linc00673 expression was higher in advanced pathologic stage, which was associated with lower miR-150-5p expression (Fig. 4d). The expression of miR-150-5p in several cell lines was showed in Fig. 4e. The efficiency of miR-150-5p mimics and inhibitors was demonstrated in Additional file 8: Figures. S6A and S6B.

We found that linc00673 level decreased significantly after transfection of miR-150-5p mimics in A549 and H1975 cells, as shown in Fig. 4f and g. Furthermore, we observed that miR-150-5p level decreased dramatically under TGF-β stimuli in A549 cells, suggesting that upregulation of linc00673 may be associated with the downregulation of miR-150-5p under TGF-β stimuli (Fig. 4h). Of note, miR-150-5p expression decreased only slightly under TGF-β stimuli in H1975 cells (Fig. 4i), which could account for the discrepant pattern of linc00673 alternation after treated with TGF-β. Meanwhile, miR-150-5p level increased significantly after linc00673 knockdown in A549 cells (Additional file 8: Figure S6C). Based on the above results, we inferred that linc00673 could be targeted by miR-150-5p and this association was important for TGF-β induced EMT process.

### Linc00673 acted as a ceRNA by sponging miR-150-5p and regulated ZEB1 expression indirectly

**Fig. 5:**
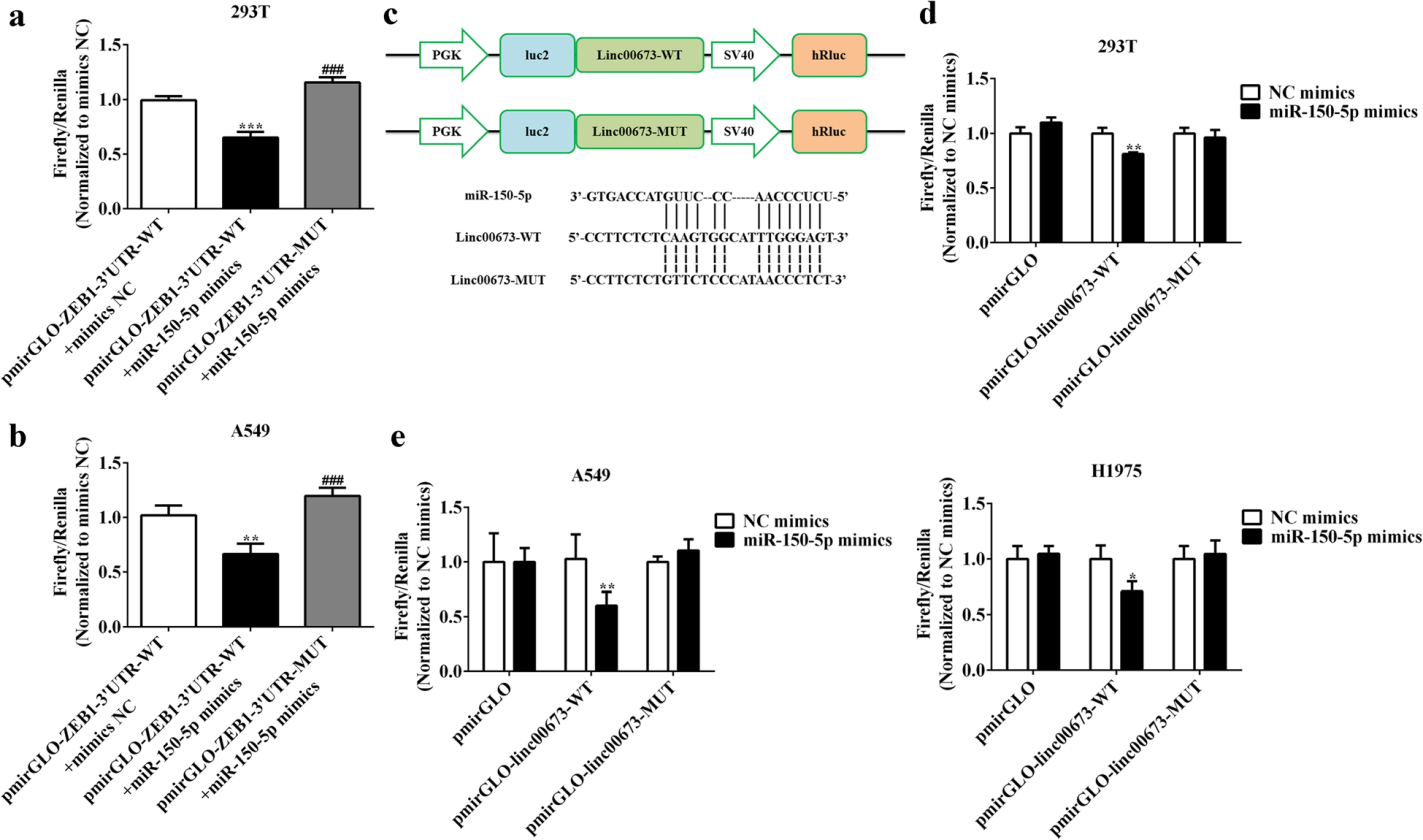
Linc00673 acted as a ceRNA by sponging miR-150-5p and regulated ZEB1 expression indirectly. **a**. 293 T cells were co-transfected with miR-150-5p mimics or mimics NC and pmirGLO-ZEB1–3’UTR-WT or pmirGLO-ZEB1–3’UTR-MUT. Luciferase activity was detected 24 h after transfection using the dual luciferase assay. **b**. A549 cells were co-transfected with miR-150-5p mimics or mimics NC and pmirGLO-ZEB1–3’UTR-WT or pmirGLO-ZEB1–3’UTR-MUT. Luciferase activity was detected 24 h after transfection using the dual luciferase assay. **c**. Predictive binding sites of miR-150-5p to linc00673 and schematic of wild-type and mutant pmirGLO-linc00673 constructs. **d**. 293 T cells were co-transfected with miR-150-5p mimics or mimics NC and pmirGLO-linc00673-WT or pmirGLO-linc00673-MUT. Luciferase activity was detected 24 h after transfection using the dual luciferase assay. **e**. A549 and H1975 cells were co-transfected with miR-150-5p mimics or mimics NC and pmirGLO-linc00673-WT or pmirGLO-linc00673-MUT. Luciferase activity was detected 24 h after transfection using the dual luciferase assay. Error bars indicate the mean ± SD. *p < 0.05, **p < 0.01, ***p < 0.005

CeRNA emerged as an important mechanism for lncRNA and miRNA regulatory network. MiR-150-5p was revealed to target ZEB1 in various cancers such colon cancer [38], esophageal squamous cancer [37] and ovarian cancer [35], and ZFAS1 was demonstrated to sequestrate miR-150-5p and exerted its pro-metastatic activity in hepatocellular cancer [39]. First, we constructed ZEB1 3’UTR wild type or mutated (predicted miR-150 binding sites) luciferase plasmids in the pmirGLO dual luciferase reporter vector, and luciferase activity was evaluated after co-transfection of miRNA and luciferase plasmids. In 293 T and A549 cells, we observed that over-expression of miR-150-5p could reduce the luciferase activity significantly, while mutation in miR-150-5p binding sites reversed luciferase activity (Fig. 5a and b). After validating that miR-150-5p could target 3’UTR of ZEB1, we then sub-cloned full-length of linc00673 (WT or MUT) into the downstream of firefly luciferase gene in the pmirGLO vector and reporter assays were performed, as shown in Fig. 5c. Compared with the control group, co-transfection with pmirGLO-linc00673-WT vector and miR-150-5p mimic reduced luciferase reporter activity significantly in 293 T, A549 and H1975 cells (Fig. 5d and e). This repressive effect was abrogated by mutations of the miR-150-5p-binding seed region in linc00673 (Fig. 5d and e). The above results suggested that linc00673 functions as a ceRNA by sponging miR-150-5p and regulated ZEB1 expression indirectly.

### Inhibition of miR-150-5p reversed linc00673 silence mediated suppressing of proliferation, migration, invasion and EMT

**Fig. 6:**
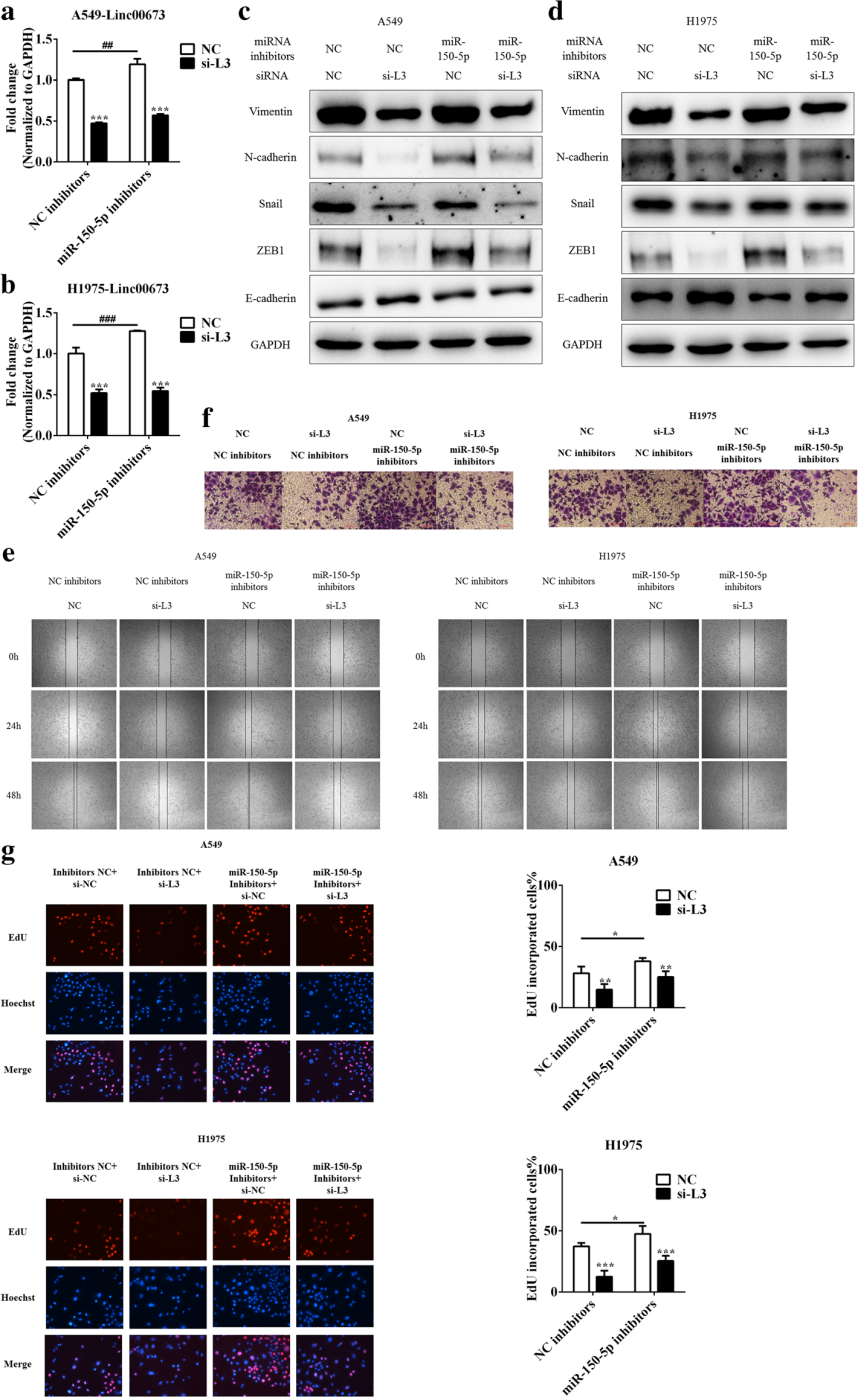
Inhibition of miR-150-5p reversed linc00673 silence mediated suppressing proliferation, migration, invasion and EMT effect. **a**. Expression of linc00673 in miR-150-5p inhibitors and si-L3 transfected A549 cells as determined by qRT-PCR. **b**. Expression of linc00673 in miR-150-5p inhibitors and si-L3 transfected H1975 cells as determined by qRT-PCR. **c**. Expression of EMT markers in miR-150-5p inhibitors and si-L3 transfected A549 cells as determined by western blot. **d**. Expression of EMT markers in miR-150-5p inhibitors and si-L3 transfected H1975 cells as determined by western blot. **e**. Wound scratch assay in miR-150-5p inhibitors and si-L3 transfected A549 and H1975 cells. **f**. Transwell invasion assay in miR-150-5p inhibitors and si-L3 transfected A549 and H1975 cells. **g**. EdU proliferation assay in miR-150-5p inhibitors and si-L3 transfected A549 and H1975 cells. Error bars indicate the mean ± SD. *p < 0.05, **p < 0.01, ***p < 0.005

To further validate the association between linc00673 and miR-150-5p, we then studied the effects of linc00673 on cell proliferation, migration, invasion and EMT after miR-150-5p was restrained. In consistent with previous results, linc00673 expression increased after transfected with miR-150-5p inhibitors while decreased after linc00673 knockdown (Fig. 6a and b). We observed that after transfected with miR-150-5p inhibitors, linc00673 silence mediated ZEB1 downregulation was partly rescued (Fig. 6c and d). Epithelial marker E-cadherin changed in accordance with ZEB1 as well. Cell migration and invasion abilities also decreased following linc00673 silencing and abrogated by miR-150-5p inhibition in A549 and H1975 cells (Fig. 6e and f). Apart from its EMT-modulatory role, ZEB1 has been reported to enhance cell proliferation in several cancers [40, 41] and miR-150-5p acted as a growth suppresser [42, 43]. The EdU proliferation assay revealed that miR-150-5p downregulation reversed linc00673 silencing mediated proliferation suppressing effect (Fig. 6g). These evidences suggested that miR-150-5p was an important mediator of linc00673 regulated proliferation, migration, invasion and EMT processes.

## Discussion

**Fig. 7:**
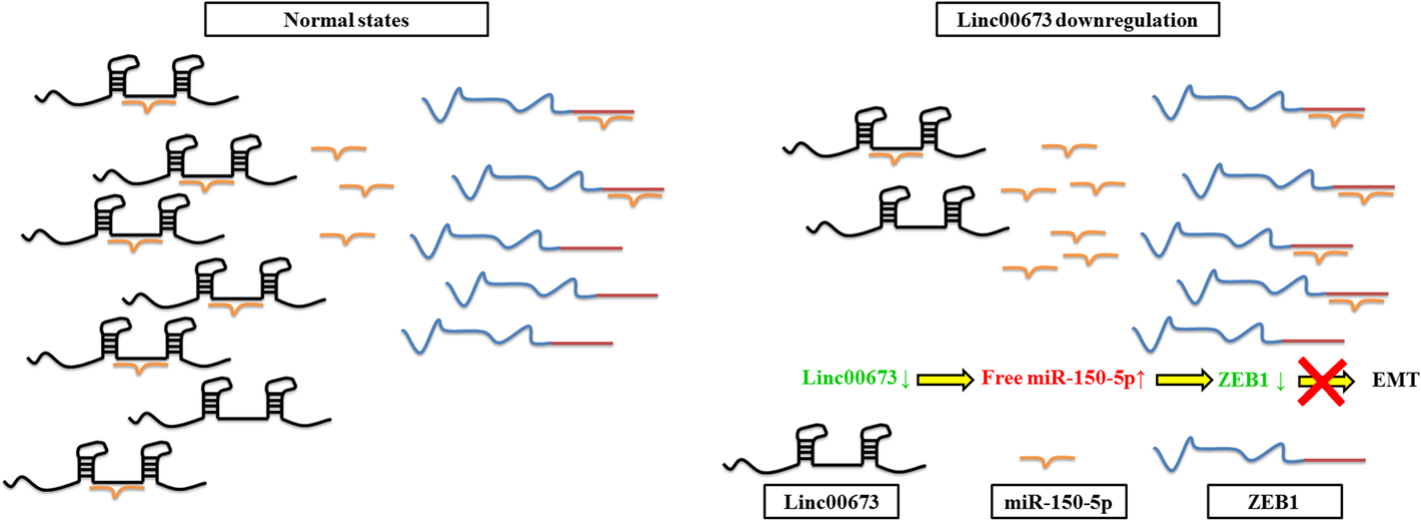
Schematic plot showed linc00673 acted as a miRNA sponge to regulate ZEB1 expression indirectly

Lung cancer is one of the major causes of cancer-related death worldwide and 5-year survival rate for NSCLC remains poor (18.2%) [9]. Researchers have focused on seeking for powerful prognostic markers such as EGFR, p53, BRCA1, k-Ras, CD44 [44–48], which would be of great value to classify patients for appropriate adjuvant therapies and predict survival outcomes precisely. In the present study, we confirmed the oncogenic role of linc00673 and identified linc00673 as an effective prognostic marker for NSCLC patients. Through in vitro and in vivo assays, we observed linc00673 could sponge miR-150-5p and modulate ZEB1 expression indirectly. Linc00673 downregulation inhibited cell proliferation, migration, invasion and EMT in NSCLC, and the correlation between linc00673 and miR-150-5p may play a crucial role in TGF-β induced EMT process (Fig. 7). Besides, although we observed promoted apoptosis after linc00673 knockdown in A549, the apoptosis level did not change in H1975 cells significantly. Thus we speculated that the role of linc00673 in promoting apoptosis may be cell line-specific, which probably was not the major function of linc00673 in NSCLC.

Linc00673 was at first identified to be significantly upregulated in NSCLC tissues compared with normal lung tissues, and Shi et al. verified that linc00673 could promote cell proliferation via interacting with the H3K4 demethylase LSD1 and suppressing NCALD expression [24, 49]. Of note, Zheng et al. reported that linc00673 expression was lower in PDAC than in normal tissues, and linc00673 inhibited PDAC cell proliferation via enhancing PTPN11–PRPF19 interaction and PTPN11 ubiquitination [23]. It seemed contradictory that linc00673 played two completely opposite roles in PDAC and NSCLC, but there were several explanations for that. First, functions of lncRNA may have tissue specificities. LncRNA H19 was reported to suppress HCC invasion and EMT via binding the hnRNP U/PCAF/RNAPol II protein complex, which was responsible for activation of miR-200 family through enhancing histone acetylation [50]. However, Han et al. observed that H19 recruited eIF4A3 and upregulated a series of cell-cycle genes, thereby promoting cell proliferation in colorectal cancer (CRC) [51]. Second, we demonstrated that linc00673 could sponge miR-150-5p in NSCLC, but whether linc00673 could acted as an effective ceRNA in PDAC remained unclear, which was mainly determined by abundance and subcellular location of ceRNA components [52]. Third, PDAC was distinct from many other cancers due to its strong invasiveness, severity of malignancy and poor prognosis [53]. However, further studies need to be carried out to comprehensively investigate this issue.

Metastasis is a primary characteristic of cancer and the leading cause of death for about 90% of cancer patients. EMT is a process during which cancer cells acquired migration ability and invasiveness, which resulted in cancer metastasis and poor prognosis [11, 14]. Repression of E-cadherin expression is an important phenotype in EMT, while ZEB1 is a major transcriptional suppressor for E-cadherin via binding to the E-box in the promoter of CDH1 gene directly [54]. Recently, several miRNAs such as miR-200 family and miR-150-5p were confirmed to target ZEB1 and caused mRNA degradation, thus blocking EMT [35–38]. As another type of ncRNA, lncRNAs are emerging as important regulators in various biological and disease development processes [2]. A multitude of lncRNAs have been verified to regulate EMT, despite that diverse mechanisms were involved. Fan et al. suggested MALAT1 repressed E-cadherin expression via associating with one subunit of polycomb repressive complex (PRC2), Suz12 [19], and high expression of MALAT1 in cancer tissues predicted worse survival for bladder cancer patients consistently. In some cases, lncRNAs harbored miRNA response elements (MREs) and acted as endogenous miRNA sponge, thus modulating de-repression of miRNA’s target gene and influencing post-transcriptional regulation. For example, Yuan et al. found that lncRNA-ATB could be activated by TGF-β and increased hepatocellular carcinoma (HCC) cell invasion via binding the miR-200 family and upregulating ZEB1 and ZEB2, thus promoting EMT [18]. LncRNA ZFAS1 promoted HCC metastatic progression by directly binding miR-150-5p and abrogated miR-150-5p mediated ZEB1 expression inhibition [39]. Therefore, identification of EMT-associated lncRNAs seemed promising and these lncRNAs may become candidates of potential prognostic biomarkers.

## Conclusions

We found that high linc00673 expression predicted worse overall survival for NSCLC patients, and linc00673 knockdown reversed TGF-β induced epithelial mesenchymal transition by sponging miR-150-5p and modulate ZEB1 expression indirectly. In addition, miR-150-5p inhibition abrogated linc00673 silencing mediated proliferation, migration, invasion and EMT suppressing effect. We validated linc00673 as an oncogene in NSCLC and revealed the molecular mechanism by which it promotes NSCLC development, which advances our understanding of its clinical significance.

## Additional files

Additional file 1: Table S1. siRNA sequences. (DOCX 15 kb)

Additional file 2: Table S2. Primers for qRT-PCR. (DOCX 15 kb)

Additional file 3: Figure S1. Kaplan-Meier survival curve for linc00673 expression in NSCLC patients. Cutpoint was set at median value. (TIFF 682 kb)

Additional file 4: Figure. S2. Effects of linc00673 on lung cancer cell apoptosis, viability, migration and invasion. (A) Expression of PARP and pro-Caspase 3 in si-NC or si-L3 transfected H1975 cells. (B) CCK-8 proliferation assay in si-NC or si-L3 transfected H520 and BEAS-2B cells. (C) Clonogenic assay in si-NC or si-L3 transfected A549 and H1975 cells. Error bars indicate the mean ± SD. *p < 0.05, **p < 0.01, ***p < 0.005. (TIFF 7639 kb)

Additional file 5: Figure. S3. In vivo images of tumor growth in NOD/SCID mice after tail vein injection of transfected A549 cells. (TIFF 3067 kb)

Additional file 6: Figure S4. Linc00673 was required for epithelial mesenchymal transition. (A) Expression of Vimentin, N-cadherin, Snail, ZEB1 and E-cadherin in TGF-β treated H1975 cells as determined by western blot. (B) Expression of Vimentin and E-cadherin in TGF-β receptor antagonist SB431542 and TGF-β treated H1975 cells as determined by western blot. (C) Morphology of si-NC or si-L3 transfected followed by TGF-β treated A549 and H1975 cells. (D) Expression of EMT markers in pcDNA3.1-linc00673 transfected H1703 cells. (E) Expression of Vimentin and E-cadherin in TNF-α treated A549 cells as determined by western blot. (F) Expression of Vimentin and E-cadherin in si-NC or si-L3 transfected followed by TNF-α treated A549 cells as determined by western blot. (G) Expression of linc00673 in TNF-α treated A549 cells as determined by qRT-PCR. (H) Immunofluorescence staining of Vimentin expression in pcDNA3.1-linc00673 transfected H1703 cells. Error bars indicate the mean ± SD. *p < 0.05, **p < 0.01, ***p < 0.005. (TIFF 41480 kb)

Additional file 7: Figure S5. Kaplan-Meier survival curve for miR-150 expression in NSCLC patients. Cutpoint was set at 53%. (TIFF 688 kb)

Additional file 8: Figure S6. Reciprocal correlation between linc00673 and miR-150-5p. (A) Expression of miR-150-5p in miR-150-5p mimics or inhibitors transfected A549 cells as determined by qRT-PCR. (B) Expression of miR-150-5p in miR-150-5p mimics or inhibitors transfected H1975 cells as determined by qRT-PCR. (C) Expression of miR-150-5p in si-NC or si-L3 transfected A549 cells as determined by qRT-PCR. (D) Error bars indicate the mean ± SD. *p < 0.05, **p < 0.01, ***p < 0.005. (TIFF 13170 kb)

## Abbreviations

ceRNA: Competing endogenous RNA
EMT: Epithelial mesenchymal transition
H3K4: histone H3 lysine 4
HCC: Hepatocellular carcinoma
lncRNAs: Long non-coding RNAs
miRNAs: microRNAs
MREs: miRNA response elements
ncRNAs: Non-coding RNAs
NSCLC: Non-small cell lung cancer
OS: Overall survival
PDAC: Pancreatic ductal adenocarcinoma
PRC2: Polycomb repressive complex
SCLC: Small cell lung cancer
SD: Standard deviation
UTRs: Untranslated regions
