# Erratum to: Long non-coding RNA linc00673 regulated non-small cell lung cancer proliferation, migration, invasion and epithelial mesenchymal transition by sponging miR-150-5p

**DOI:** 10.1186/s12943-017-0716-6

**Published:** 2017-08-29

**Authors:** Wei Lu, Honghe Zhang, Yuequn Niu, Yongfeng Wu, Wenjie Sun, Hongyi Li, Jianlu Kong, Kefeng Ding, Han-Ming Shen, Han Wu, Dajing Xia, Yihua Wu

**Affiliations:** 1 Department of Toxicology, Zhejiang University School of Public Health, 866 Yuhangtang Road, Hangzhou, People’s Republic of China; 2 Department of Surgical Oncology, Second Affiliated Hospital, Zhejiang University School of Medicine, Hangzhou, People’s Republic of China; 3 Department of Pathology, Zhejiang University School of Medicine, Hangzhou, People’s Republic of China; 4 Department of Physiology, Yong Loo Lin School of Medicine, National University of Singapore, Singapore, Singapore; 5 Department of Ophthalmology, Second Affiliated Hospital, Zhejiang University School of Medicine, Hangzhou, People’s Republic of China

## Erratum

After the publication of this work [1] an error was noticed in Additional file 1: Table S1 regarding the siRNA sequences, in which two siRNAs (si-L4/L5) were omitted from the figure. The updated version (now correctly added) has the sequences of si-L1/L2/L3 and the other two, si-L4/L5. Since the last two siRNAs (si-L4/L5) served as further confirmation for the original data, this update does not affect the findings or conclusions of the article. Nevertheless, we apologize for the inconvenience.

## Additional file

Additional file 1: siRNA sequences. (DOCX 15 kb)

## References

[1] Lu W, Zhang H, Niu Y, Wu Y, Sun W, Li H (2017). Long non-coding RNA linc00673 regulated non-small cell lung cancer proliferation, migration, invasion and epithelial mesenchymal transition by sponging miR-150-5p. Mol Cancer.

