# Supplementary material for: Long non-coding RNA linc00673 regulated non-small cell lung cancer proliferation, migration, invasion and epithelial mesenchymal transition by sponging miR-150-5p
Source: Mol Cancer. 2017 Jul 11;16:118. doi: 10.1186/s12943-017-0685-9 (PMC5504775; doi:10.1186/s12943-017-0685-9)
Supplement: Supplementary file 1 — siRNA sequences. (DOCX 15 kb) [file 12943_2017_685_MOESM1_ESM.docx]

| siRNA sequences | |
| --- | --- |
| NC-sense | UUCUCCGAACGUGUCACGUTT |
| NC-antisense | ACGUGACACGUUCGGAGAATT |
| si-L1-sense | GCUUUCUACCACACCCUUUTT |
| si-L1-antisense | AAAGGGUGUGGUAGAAAGCTT |
| si-L2-sense | CCUUGGAAUAGUAACUCUUTT |
| si-L2-antisense | AAGAGUUACUAUUCCAAGGTT |
| si-L3-sense | GGAGUCCAUGCCAGAUCAUTT |
| si-L3-antisense | AUGAUCUGGCAUGGACUCCTT |
