# Supplementary material for: Long non-coding RNA linc00673 regulated non-small cell lung cancer proliferation, migration, invasion and epithelial mesenchymal transition by sponging miR-150-5p
Source: Mol Cancer. 2017 Jul 11;16:118. doi: 10.1186/s12943-017-0685-9 (PMC5504775; doi:10.1186/s12943-017-0685-9)
Supplement: Supplementary file 2 — Primers for qRT-PCR. (DOCX 15 kb) [file 12943_2017_685_MOESM2_ESM.docx]

| Primers for qRT-PCR | |
| --- | --- |
| Linc00673-F | CCGTGTAAAGAGGCCAGTGT |
| Linc00673-R | ACACGAGCCTTCACCATCAG |
| E-cadherin-F | CAAGCTATCCTTGCACCTCAG |
| E-cadherin-R | GCATCAGAGAACTCCTATCTTG |
| Vimentin-F | GGACCAGCTAACCAACGACA |
| Vimentin-R | AAGGTCAAGACGTGCCAGAG |
| Snail-F | CTCGGACCTTCTCCCGAATG |
| Snail-R | AAAGTCCTGTGGGGCTGATG |
| ZEB1-F | AAGTGGCGGTAGATGGTAATGT |
| ZEB1-R | AAGGAAGACTGATGGCTGAAAT |
| GAPDH-F | CACCCACTCCTCCACCTTTG |
| GAPDH-R | CCACCACCCTGTTGCTGTAG |
| miR-150-5p 3’ specific primer | TCTCCCAACCCTTGTACCAGTG |

MiRNA specific 5’ primer, U6-Forward and U6-Reverse were provided by the Mir-X™ miRNA First Strand Synthesis Kit
