# Supplementary figures and images for: Long non-coding RNA linc00673 regulated non-small cell lung cancer proliferation, migration, invasion and epithelial mesenchymal transition by sponging miR-150-5p
Source: Mol Cancer. 2017 Jul 11;16:118. doi: 10.1186/s12943-017-0685-9 (PMC5504775; doi:10.1186/s12943-017-0685-9)

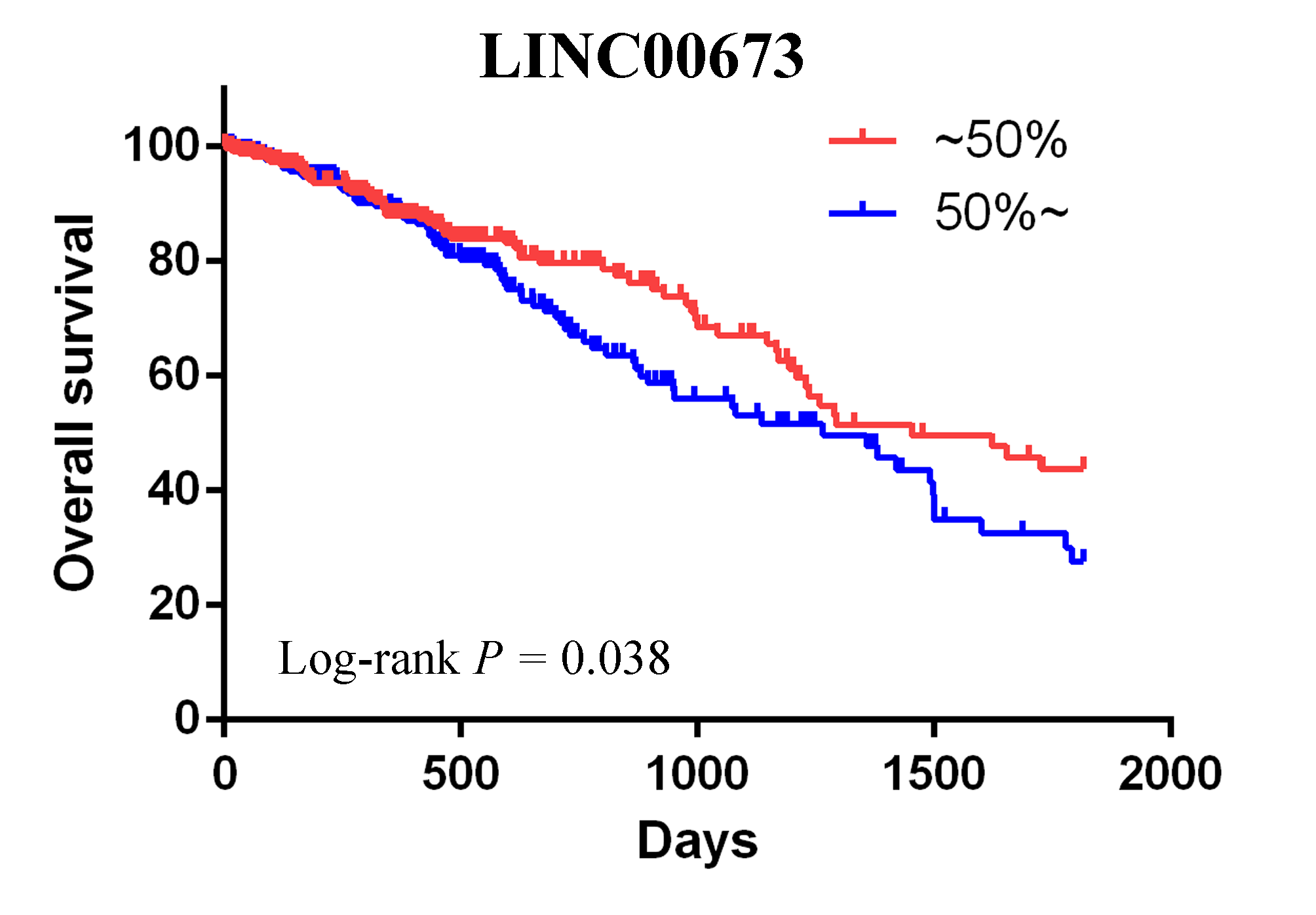

Supplement: Supplementary file 3 — Kaplan-Meier survival curve for linc00673 expression in NSCLC patients. Cutpoint was set at median value. (TIFF 682 kb) [file 12943_2017_685_MOESM3_ESM.tif]

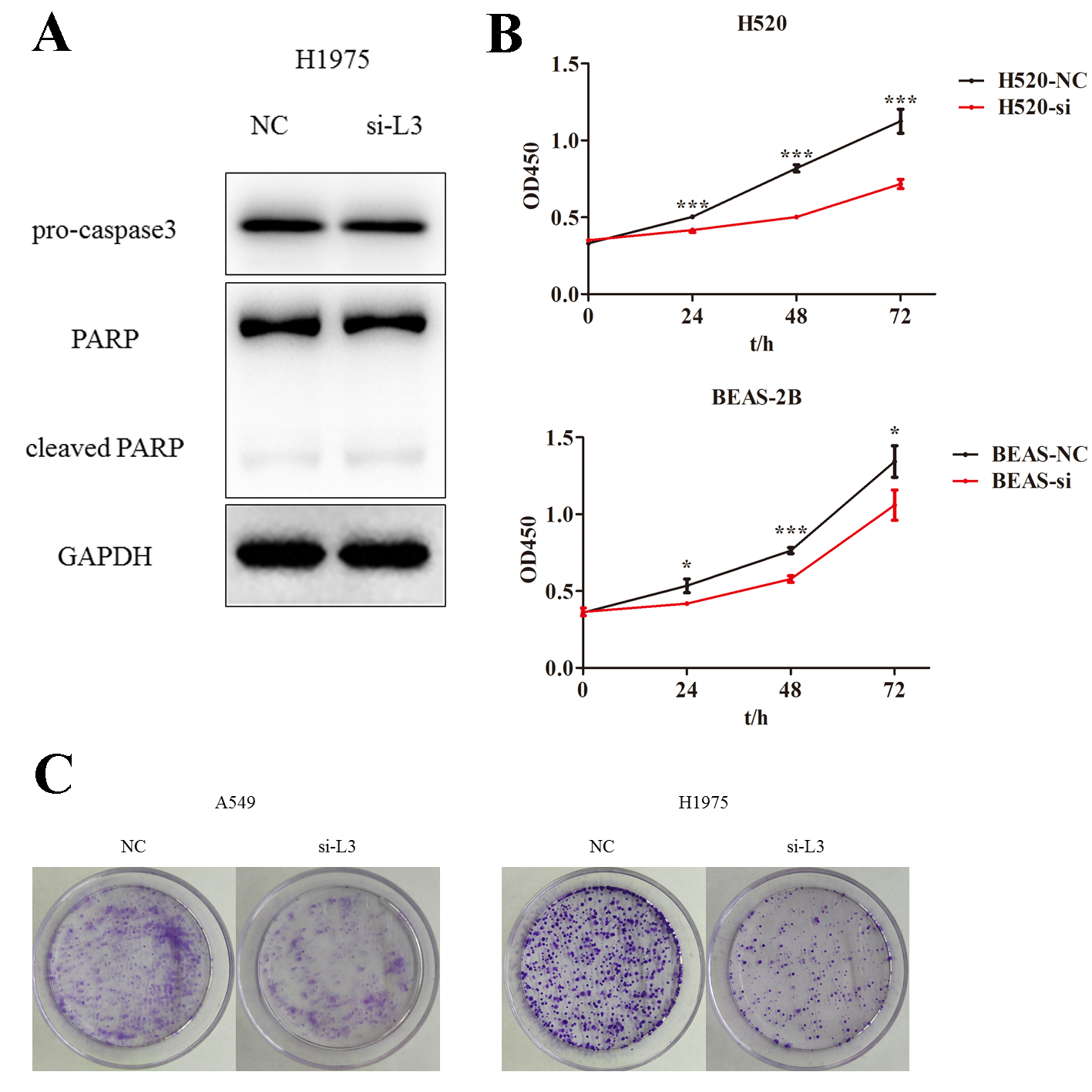

Supplement: Supplementary file 4 — Effects of linc00673 on lung cancer cell apoptosis, viability, migration and invasion. (A) Expression of PARP and pro-Caspase 3 in si-NC or si-L3 transfected H1975 cells. (B) CCK-8 proliferation assay in si-NC or si-L3 transfected H520 and BEAS-2B cells. (C) Clonogenic assay in si-NC or si-L3 transfected A549 and H1975 cells. Error bars indicate the mean ± SD. *p < 0.05, **p < 0.01, ***p < 0.005. (TIFF 7639 kb) [file 12943_2017_685_MOESM4_ESM.tif]

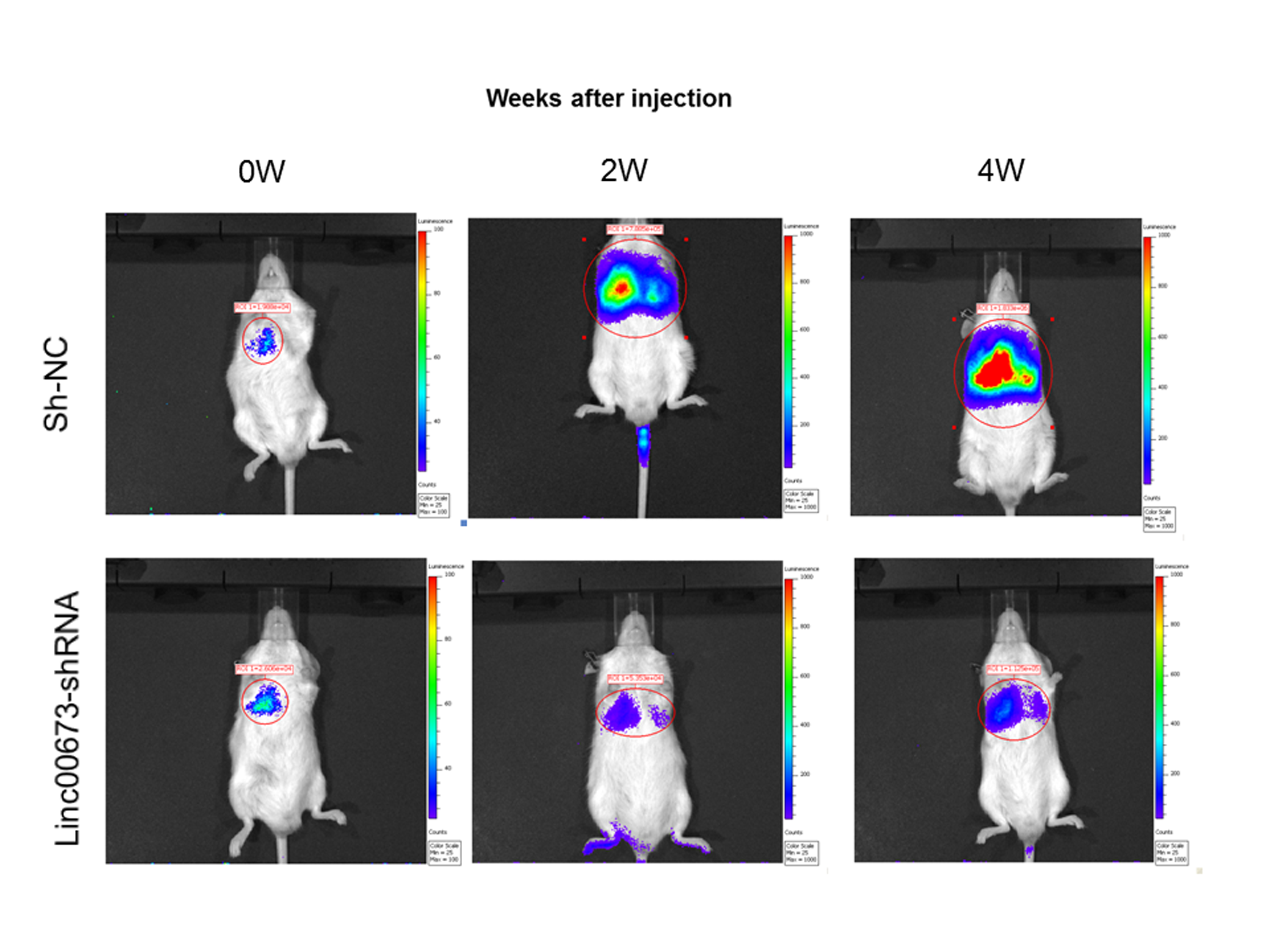

Supplement: Supplementary file 5 — In vivo images of tumor growth in NOD/SCID mice after tail vein injection of transfected A549 cells. (TIFF 3067 kb) [file 12943_2017_685_MOESM5_ESM.tif]

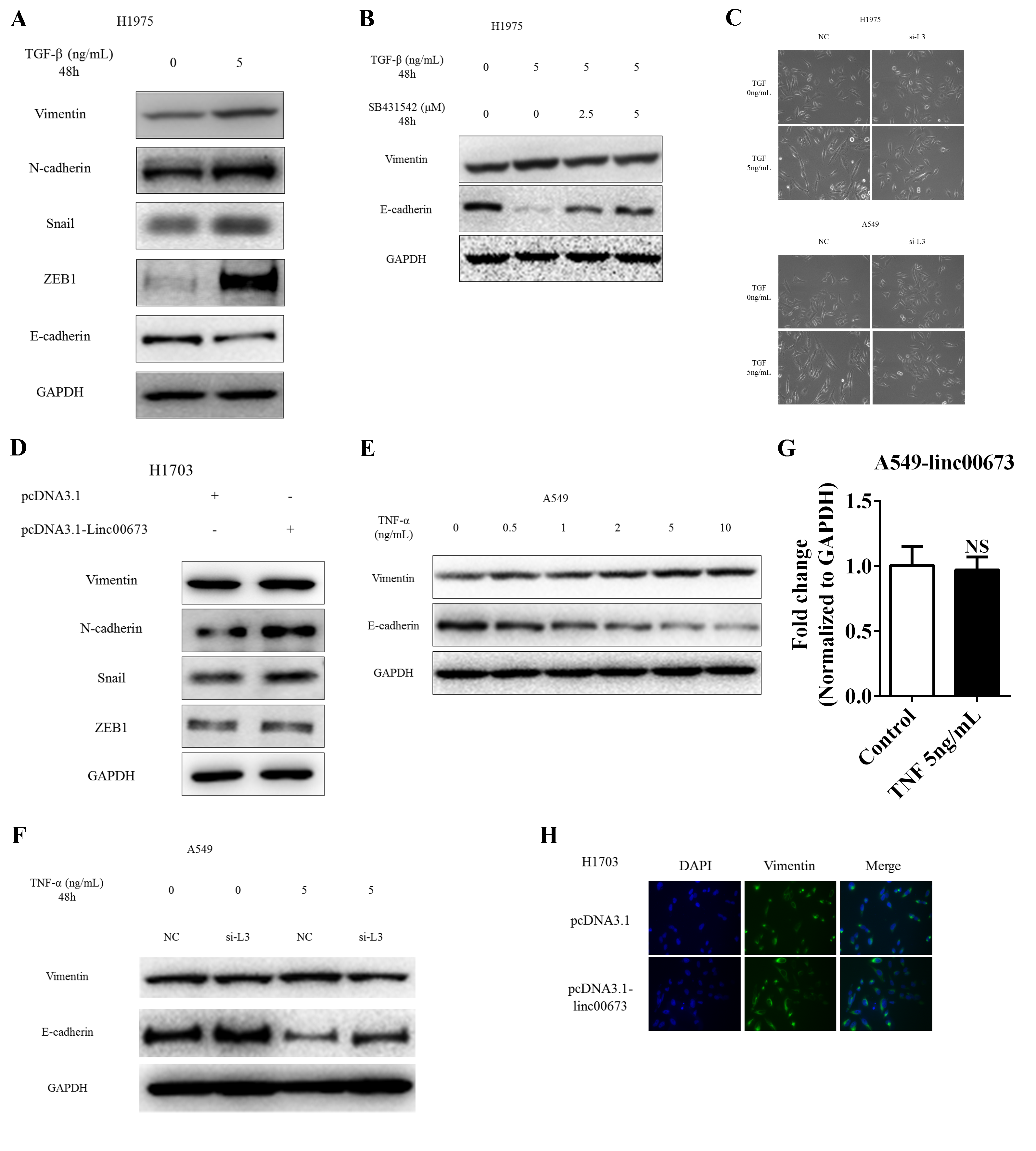

Supplement: Supplementary file 6 — Linc00673 was required for epithelial mesenchymal transition. (A) Expression of Vimentin, N-cadherin, Snail, ZEB1 and E-cadherin in TGF-β treated H1975 cells as determined by western blot. (B) Expression of Vimentin and E-cadherin in TGF-β receptor antagonist SB431542 and TGF-β treated H1975 cells as determined by western blot. (C) Morphology of si-NC or si-L3 transfected followed by TGF-β treated A549 and H1975 cells. (D) Expression of EMT markers in pcDNA3.1-linc00673 transfected H1703 cells. (E) Expression of Vimentin and E-cadherin in TNF-α treated A549 cells as determined by western blot. (F) Expression of Vimentin and E-cadherin in si-NC or si-L3 transfected followed by TNF-α treated A549 cells as determined by western blot. (G) Expression of linc00673 in TNF-α treated A549 cells as determined by qRT-PCR. (H) Immunofluorescence staining of Vimentin expression in pcDNA3.1-linc00673 transfected H1703 cells. Error bars indicate the mean ± SD. *p < 0.05, **p < 0.01, ***p < 0.005. (TIFF 41480 kb) [file 12943_2017_685_MOESM6_ESM.tif]

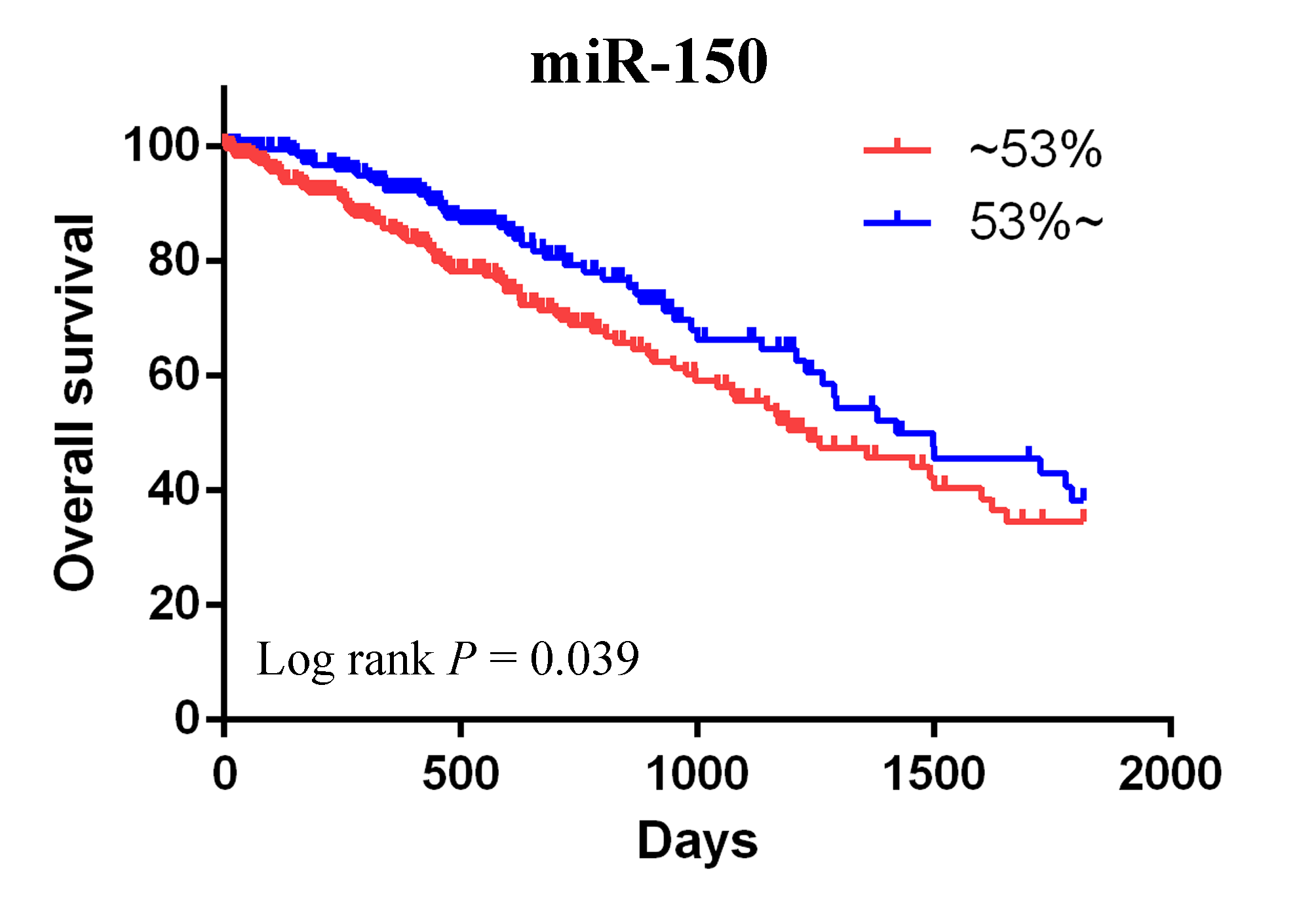

Supplement: Supplementary file 7 — Kaplan-Meier survival curve for miR-150 expression in NSCLC patients. Cutpoint was set at 53%. (TIFF 688 kb) [file 12943_2017_685_MOESM7_ESM.tif]

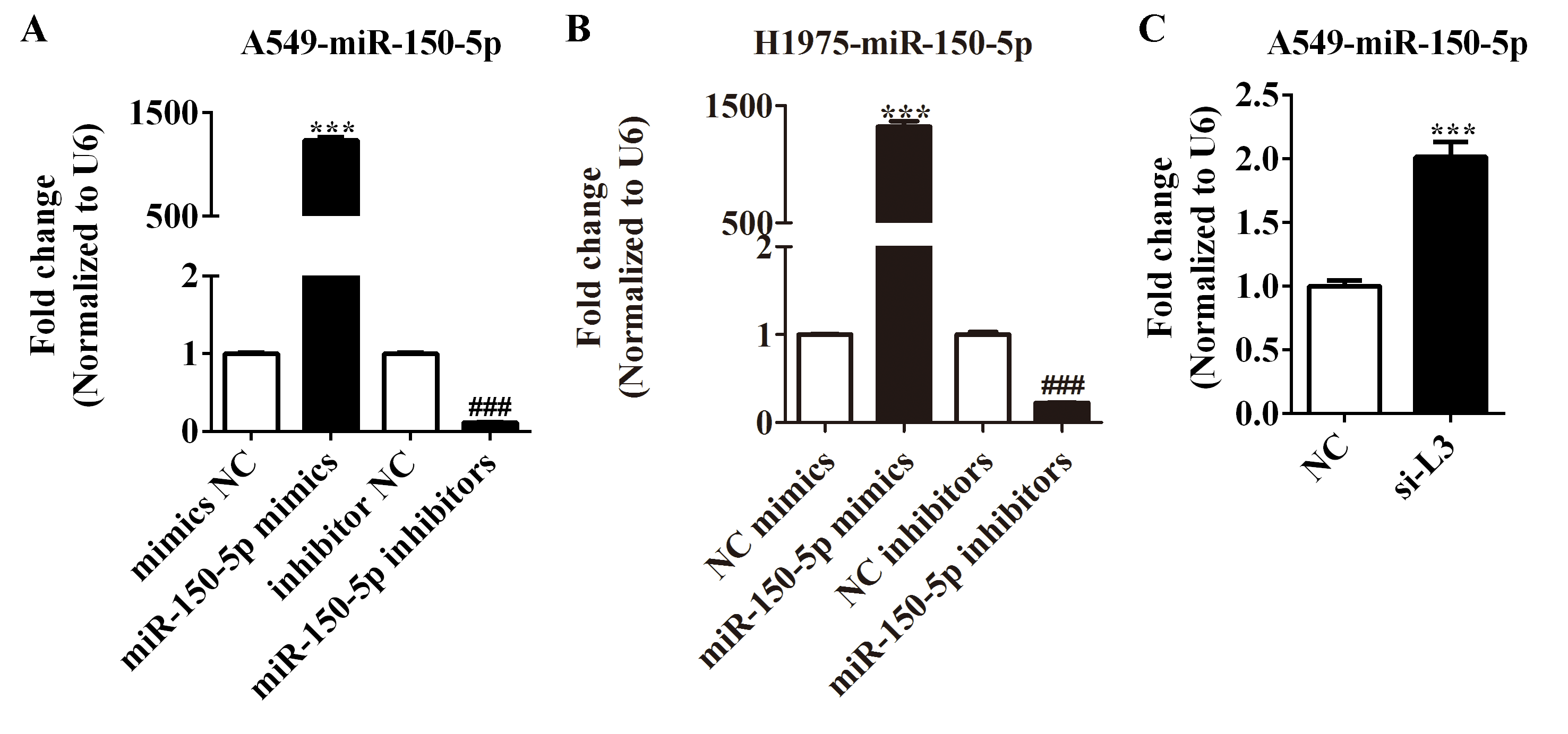

Supplement: Supplementary file 8 — Reciprocal correlation between linc00673 and miR-150-5p. (A) Expression of miR-150-5p in miR-150-5p mimics or inhibitors transfected A549 cells as determined by qRT-PCR. (B) Expression of miR-150-5p in miR-150-5p mimics or inhibitors transfected H1975 cells as determined by qRT-PCR. (C) Expression of miR-150-5p in si-NC or si-L3 transfected A549 cells as determined by qRT-PCR. (D) Error bars indicate the mean ± SD. *p < 0.05, **p < 0.01, ***p < 0.005. (TIFF 13170 kb) [file 12943_2017_685_MOESM8_ESM.tif]
